# A single design strategy for dual sensitive pH probe with a suitable range to map pH in living cells

**DOI:** 10.1038/srep15540

**Published:** 2015-10-21

**Authors:** Kang-Kang Yu, Ji-Ting Hou, Kun Li, Qian Yao, Jin Yang, Ming-Yu Wu, Yong-Mei Xie, Xiao-Qi Yu

**Affiliations:** 1 Key Laboratory of Green Chemistry and Technology, Ministry of Education, College of Chemistry, Sichuan University, No. 29, Wangjiang Road, Chengdu, 610064, P.R. China; 2 State Key Laboratory of Biotherapy/Collaborative Innovation Center for Biotherapy, Sichuan University, No. 17, People’s South Road, Chengdu, 610041, P.R. China

## Abstract

Due to the lack of a proper imaging approach, a veracious pH map of normal and abnormal cell is still rare. In this work, we presented a rhodamine-salicylaldehyde combination (**Rh-SA2**) as a novel pH probe, which has dual sensitive units for both acidic and basic environment. This dual sensitive probe acts like a chameleon in living cells and offers the doubling guarantees for endocellular pH mapping. Moreover, a quantitative measurement of cellular pH changes was allowed and the endocellular pH values under drug-associated stimuli were also investigated.

In cells, the endocellular pH distribution is uneven with the unique functions of organelles determining the ambient pH value of each individual compartment^1^. For example, endosomes and lysosomes are acidic^2^, the nucleus and cytochylema are close to neutral^3^,^4^, and normal mitochondria is notably alkaline^5^,^6^,^7^,^8^. Abnormal pH changes in the cells can cause severe harm, such as detrimental effects on membrane contractility, the generation of toxic free radicals, cellular necrosis and apoptosis, etc.^9^,^10^,^11^,^12^,^13^. Not surprisingly, the proton gradient across endocellular compartment membranes and pH homeostasis in the surrounding media have profound significance on cellular proliferation, function, and survival^14^,^15^,^16^,^17^. Therefore, the development of a method for the quantitative detection of alterations in endocellular pH homeostasis under external stimuli, as well as the different endocellular proton gradients, is urgently needed in order to gain a cellular-level understanding of disease pathogenesis.

For achieving the visualized mapping of heterogeneous pH in living cells and quantification of endocellular pH values, fluorescence imaging stands out as an ideal option since this is a minimally invasive and dynamic imaging procedure^18^,^19^,^20^,^21^,^22^,^23^. More importantly, compared with other imaging methods (optical coherence tomography, electron microscopy and magnetic resonance imaging), fluorescence microscopy can both distinguish between various cellular organelles and trace endocellular biomolecules (proteins, DNA and RNA)^22^. To devise a remarkable fluorescent probe for mapping endocellular pH and quantifying variations in endocellular pH, the following prerequisites need to be fulfilled: 1) the fluorescence intensity of probe should vary dramatically within the appropriate pH range (pH 4.5–8.0); 2) the fluorescent pH probe used confocal microscopic bioimaging should rely on ratiometric fluorescence from two different exciting light sources, as this strategy allows for self-calibration to effectively eliminate interference; 3) a single pH probe should be “dual sensitive”, possessing one component for the detection of protons and another for hydroxyl ions. In regard to this last requirement, a pH probe with a single ionizable functional group would be more prone to interference and inaccurate pH mapping/detection due to the complicated endocellular chemical milieu. A dual-sensitive probe provides “double insurance”, increasing the precision of both qualitative imaging and quantitative measurement. To the best of our knowledge, despite extensive reports on many diverse pH fluorescent pH probes^24^,^25^,^26^,^27^,^28^,^29^,^30^,^31^,^32^, a compound that satisfies all three of our stated requirements is still remarkably rare^33^,^34^,^35^. Herein, we would like to report the development of a novel fluorescent probe which meets all of our criteria, thus allowing for the facile and accurate mapping and detection of pH in living cells.

Rhodamine possesses many beneficial properties as a biocompatible fluorescent probe including good photostability, excellent photo-physical properties, moderate water-solubility, *et cetera*^36^,^37^; our group previously reported some successful rhodamine-derived probes^38^,^39^. Accordingly, we selected the rhodamine framework as the hydronium sensitive portion (pK_a_ < ~5) of our new dual-sensitive probe^24^,^32^. As for the hydroxyl ion sensitive portion, we chose the salicylaldehyde framework since this moiety exhibits a unique emission band discriminant from rhodamine and it is able to provide a phenolic hydroxyl under basic condition^40^,^41^. A facile one-step conjugation of the rhodamine and salicylaldehyde moieties was employed to afford a novel dual-sensitive pH fluorescent probe which possesses two unique emission bands and can be analyzed using two discrete excitation source allow for efficient pH mapping of entire living cells and quantitative detection of pH alteration under different stimuli.

## Result

### Optical spectra properties of Rh-SA1/2/3

**Figure 1:**
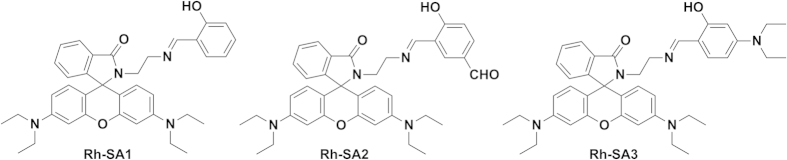
The structures of rhodamine-salicylaldehyde combinations.

To explore the impact of the electronic effects on the pH sensitivity of phenol moiety, we designed and synthesized three compounds with different substituents on the phenol ring of salicylaldehyde (Fig. 1). The structures of **Rh-SA1/2/3** and intermediates were confirmed by ^1^H NMR, ^13^C NMR and ESI-MS spectroscopy.

**Figure 2:**
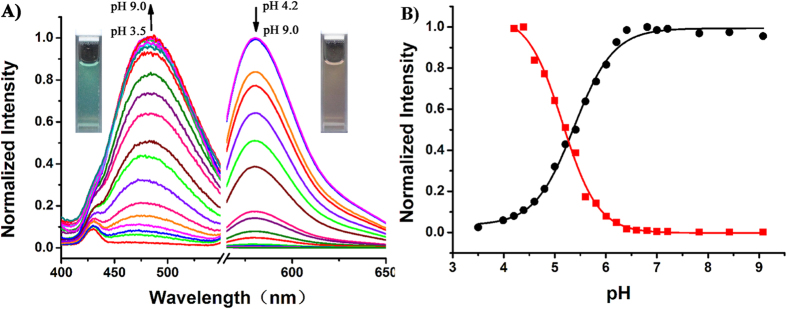
(**A**) Fluorescence emission spectral changes of **Rh-SA2** (5 μM) in B–R buffer solution at different pH values, and the maximum emission intensity was measured at 480 nm (*λ*_*ex*_ 375 nm) and 580 nm (*λ*_*ex*_ 550 nm). (**B**) Plot of normalized fluorescence intensity at 480 nm (black) and 580 nm (red) as a function of pH for **Rh-SA2**. Inset: plot of *I*_480_/*I*_580_ versus different pH values. pH 3.50, 3.99, 4.20, 4.39, 4.60, 4.80, 5.00, 5.21, 5.41, 5.60, 5.81, 6.01, 6.21, 6.41, 6.60, 6.81, 7.00, 7.21, 7.83, 8.42, and 9.08.

Initially, the fluorescence pH titrations of **Rh-SA1/2/3** were investigated in Britton–Robinson (B–R) buffer solutions. Interestingly, we found that the rhodamine portion of these three probes all have a regular and fairly good sensitivity toward protons, while the different phenolic groups display varying responses upon deprotonation; only **Rh-SA2** demonstrated an ordered fluorescence change towards both acidic and basic pH variation (Fig. 2). As for **Rh-SA1** and **Rh-SA3**, the fluorescence intensity of phenolic moiety did not have a regular enhancement upon decreasing pH (Figs S1 and S2). We suspect that the key reason that the phenolic group in **Rh-SA2** manifests a better response than **Rh-SA1** and **Rh-SA3** toward gradually alkalizing environments is the presence of electron-withdrawing formyl group (-CHO) in **Rh-SA2**. This electron-withdrawing group reduces the pK_a_ of the phenolic proton well within the biologically-relevant pH range investigated and it potentially decreases the reactivity of the resulting phenoxide to undergo undesired side reactions^42^.

**Figure 3:**
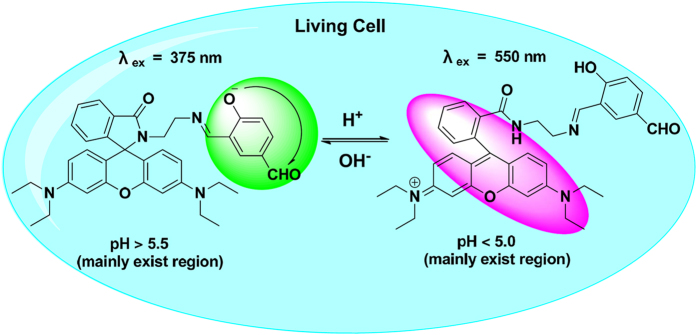
The pH mapping mechanism of Rh-SA2 in living cells.

As shown in Fig. 1, at pH 4.2 **Rh-SA2** displays a typical emission band around 580 nm (λ_ex_ 550 nm), corresponding to the opened form of the rhodamine spiro-ring unit. With an increase in pH from 4.2 to 7.0, however, the fluorescence intensity of **Rh-SA2** at 580 nm demonstrated a corresponding gradual reduction. Moreover, a new emission peak at 480 nm (λ_ex_ 375 nm), which was attributed to phenoxide anion, was observed. Simultaneously, the rising pH (4.2 to 7.0) also induced a 40-fold enhancement of fluorescence at 480 nm and a 233-fold reduction at 580 nm. Moreover, compound **Rh-SA2** exhibits bright green luminescence at pH 7.5 and orange fluorescence at pH 4.2 under ultraviolet illumination. The quantum yields of **Rh-SA2** at pH 4.0 and 7.0 were calculated to be 0.343 and 0.015 respectively (rhodamine, Table S1). The pH titration curve for **Rh-SA2** implies that under acidic conditions, protonation leads to ring-opening in the rhodamine unit with a corresponding strong orange fluorescence, whereas gradual deprotonation of the phenolic moiety in **Rh-SA2** as the solution becomes more alkaline results in a corresponding green fluorescence emission. It is worth noting that the pK_a_ values of the rhodamine and phenol units in **Rh-SA2** were calculated to be 5.19 and 5.54, respectively (Fig. S4). As mentioned above, the pH range inside an entire cell is not very wide; tiny changes in endocellular pH values can have drastic impacts on cellular metabolism and survival. The two pK_a_ values for **Rh-SA2** are definitely within the appropriate range for pH mapping of living cells and the dual sensitive channels could act like a “double insurance” to guarantee the veracity of our pH mapping efforts (Fig. 3).

### The anti-interferences of Rh-SA1/2/3

Due to the extraordinary pH sensitivity of **Rh-SA2** *in vitro*, we mainly focused on exploring the relevant properties of **Rh-SA2** in the following experiments. With the purpose of mapping endocellular pH and considering the complicated microenvironments inside living cells, several possible interfering species were examined, specifically essential cations and anions, glucose, cysteine (Cys), homocysteine (Hcy), and glutathione (GSH), at representative pH values (5.0 and 7.0). As illustrated in Fig. S5, no obvious spectroscopic changes were detected in either emission band under all of the conditions, indicating that **Rh-SA2** exhibits high selectivity and can be used to monitor pH variations in living cells without significant interference.

### Cytotoxicity and fluorescence imaging in living cells

**Figure 4:**
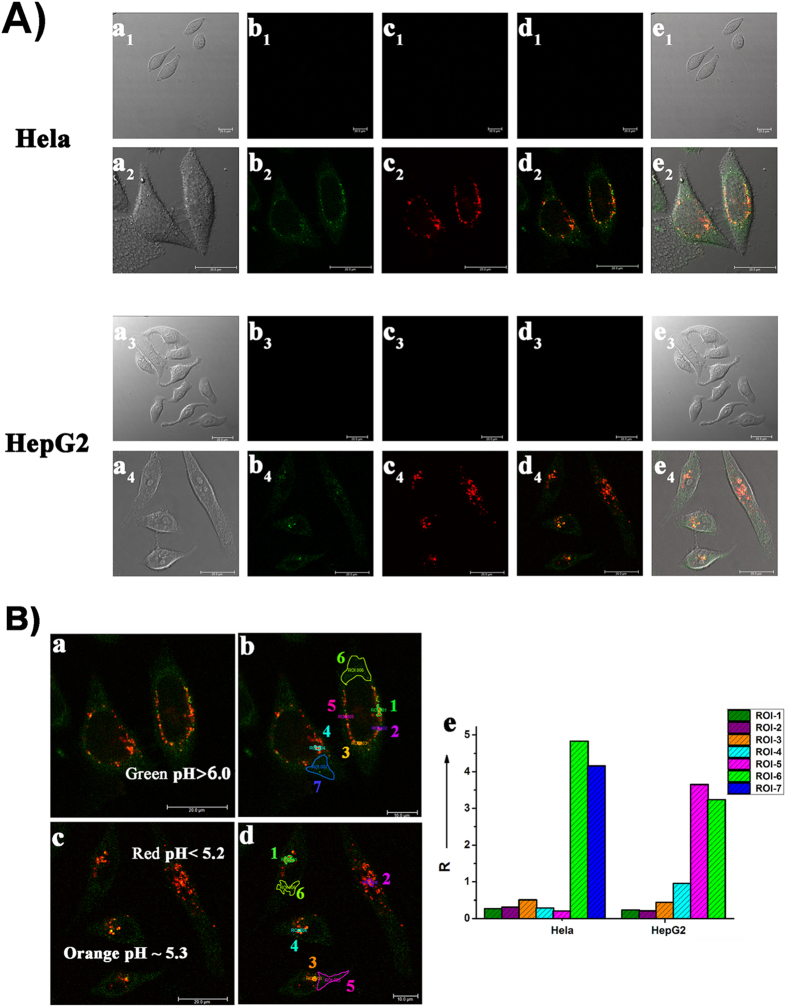
Confocal microscopy images of HeLa and HepG2 cells. Cell images were collected at 420–500 nm for the green channel (λ_ex_ 405 nm) and 565–650 nm for the red channel (λ_ex_ 550 nm). (**A**) a_1_-e_1_ and a_3_-e_3_: control group of untreated cells; a_2_-e_2_ and a_4_-e_4:_ cells treated with **Rh-SA2** (5 μM). (**B**) a and b are the image of **Rh-SA2** in HeLa cells, and seven regions of intrest (ROI) are selected (ROI 1, ROI 2, ROI 4 and ROI 5: different shades of red, ROI 3: orange, ROI 6 and ROI 7: different shades of green regions); c and d are the image of **Rh-SA2** in HepG2 cells, and six regions of intrest (ROI) are selected (ROI 1 and ROI 2: different shades of red, ROI 3 and ROI 4: different shades of orange, ROI 5 and ROI 6: different shades of green regions); the coordinate graph e shows the ratio of fluorescence in the two channel (R = *I*_green_/*I*_red_) for the different ROIs.

A standard CCK-8 assay showed that **Rh-SA2** exhibited low cytotoxicity (Fig. S6). Encouraged by this result, we conducted confocal microscopy experiments on both HeLa and HepG2 cells incubated with **Rh-SA2** (5 μM) for 30 min. As Fig. 4A suggests, **Rh-SA2** could cross the cell membrane efficiently, thus avoiding the need for any other loading methods like electroporation or microinjection, both of which would do harm to the living cells. More importantly, the dual channels for the two different sensitive parts of **Rh-SA2** indeed display their own staining results, as expected. Upon excitation at 405 nm (green channel, the phenoxide of **Rh-SA2**), various shades of green light were observed throughout the entire cytochylema; upon excitation at 552 nm (red channel, the rhodamine part of **Rh-SA2**), numerous red spots within the cells appeared, presumably within some acid vesicular organelles. By overlaying the green and red channels, independent green regions and red spots were observed in addition to certain orange flecks in distinct domains of the living cells. Based on these results, we can obtain an endocellular pH map (Fig. 4B).

Combining the two pK_a_ values for **Rh-SA2**, it is possible to qualitatively estimate the pH values of these different color zones: green areas have a pH value more than 6.0, the pH values of red spots are less than 5.2, and the pH of orange-colored spots values are about 5.3. With this framework in mind, a semi-quantitative analysis of the cells was conducted. Specifically, the ratios of fluorescent intensity between the green channel and red channel was determined for some regions of interest (ROIs) in the HeLa and HepG2 cells based on the coordinate graph illustrated in Fig. 4. As we predicted, the green/red ratio gradually reduced from the green regions (ROI 6 & 7 of HeLa cells, ROI 5 & 6 of HepG2 cells) to the orange-colored areas (ROI 3 of HeLa cells, ROI 3 & 4 of HepG2 cells) and, finally, red spots (ROI 1, 2, 4 & 5 of HeLa cells, ROI 1 & 2 of HepG2 cells), indicating that the pH values of each area was also decreasing in turn. Moreover, the ratios are slightly different for the various shades of the same color. All of the above results reveal that in the living cells **Rh-SA2** could act like a chameleon, which is quite sensitive to the surrounding environment and presents a comprehensiveness pH map.

### Lysosome-specific fluorescence imaging in living cells and drug stimulus

Several reports on related rhodamine derivatives indicate that this fluorophore effectively stains lysosomes^11^. To explore whether the endocellular acidic vesicular organelles illuminated as red spots with **Rh-SA2** are also lysosomes, we carried out colocalization experiments combining **Rh-SA2** and commercial lysosome-specific fluorescent dyes (Fig. S7). The red channel of **Rh-SA2** matches well with the green channel of LysoTracker Green (LTG) in both HeLa (Pearson’s correlation, 0.84) and HepG2 cells (Pearson’s correlation, 0.85), suggesting that the protonated ring-opened form of rhodamine **Rh-SA2** accumulated in the lysosomes of living cells.

**Figure 5:**
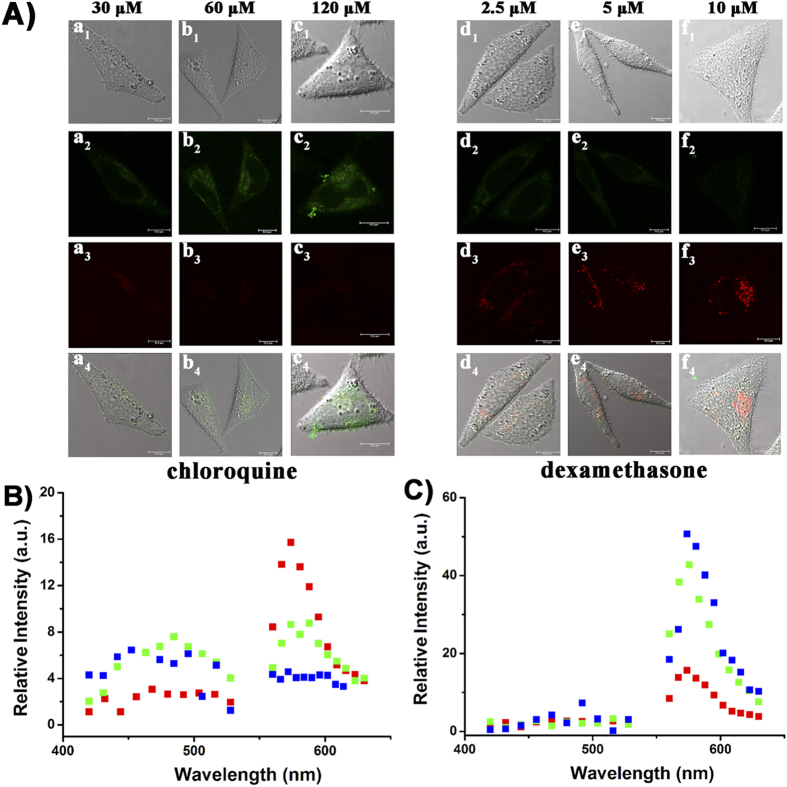
(**A**) Concentration dependent chloroquine and dexamethasone induced endocellular pH variations of HeLa cells. The cells were incubated with 5 μM **Rh-SA2** for 30 min at 37 °C in the presence of different concentration of chloroquine and dexamethasone, respectively. All the cell images were collected at 420–530 nm for the green channel (λ_ex_ 405 nm) and 565–650 nm for the red channel (λ_ex_ 550 nm). (**B**) Fluorescence spectra of the HeLa cells exposed to varying concentrations of chloroquine. Red, green, and blue indicate concentrations of 30, 60, and 120 μM, respectively. (**C**) Fluorescence spectra of the HeLa cells exposed to varying concentrations of dexamethasone. Red, green, and blue indicate concentrations of 2.5, 5.0, and 10.0 μM, respectively.

Nevertheless, to be an excellent endocellular pH mapping probe, it is not enough to present a pH map of normal cells. Therefore, **Rh-SA2** was applied toward sensing endocellular pH variations induced via different drugs. Chloroquine, an antimalarial drug, is known to induce an increase in lysosomal pH. Alternatively, dexamethasone, an anti-inflammatory drug, is known to induce cellular acidification and apoptosis^43^,^44^,^45^,^46^,^47^. Accordingly, these two drugs were selected as appropriate stimuli to inspect whether **Rh-SA2** is capable of providing an exact endocellular pH map under abnormal circumstances. As shown in Fig. 5, an increase in the concentration of chloroquine resulted in a corresponding brightening in the green channel fluorescence and weakening of the red channel fluorescent intensity. An alternate situation was observed upon increase the concentration of dexamethasone; the fluorescence of the red channel grew brighter as the green light faded. A dual-channel spectral analysis of HeLa cells stained with **Rh-SA2** provided more information about the fluorescent intensity changes upon addition of either of these two drugs. Combined, these results demonstrate that **Rh-SA2** not only can afford high-efficiency pH mapping of normal living cells, but it also has the capacity for pH mapping of abnormal cells under external stimuli.

### Intracellular pH calibration

**Figure 6:**
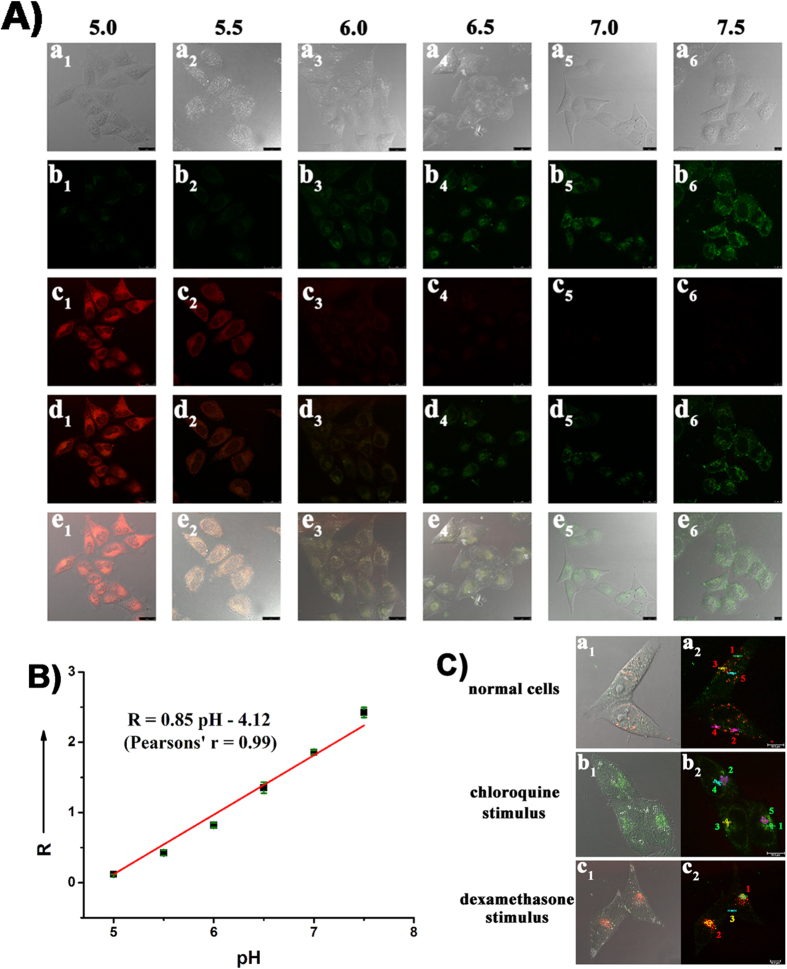
(**A**) Confocal microscopy images of HeLa cells clamped at pH 5.0, 5.5, 6.0, 6.5, 7.0, and 7.5, **Rh-SA2** (5 μM). All the cell images were collected at 420–530 nm for the green channel (λ_ex_ 405 nm) and 565–650 nm for the red channel (λ_ex_ 550 nm). (**B**) Intracellular pH calibration curve of **Rh-SA2** in HeLa cells. R means the ratio of green to red fluorescence intensity of each cell (*I*_green_/*I*_red_). (**C**) Confocal microscopy images of: a_1_ and a_2_, **Rh-SA2** (5 μM) loaded HeLa cells; b_1_ and b_2_, **Rh-SA2** (5 μM) treated living Hela cells in the presence of 60 μM chloroquine ; c_1_ and c_2_, **Rh-SA2** (5 μM) treated living Hela cells in the presence of 5 μM dexamethasone. And some ROIs are given to quantitatively calculate their pH values.

**Figure 7:**
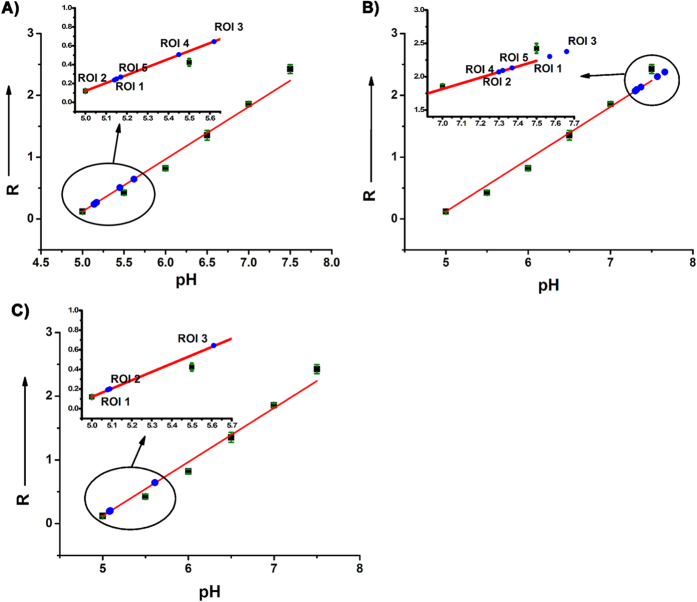
Intracellular pH calibration curve of 5 μM Rh-SA2 in HeLa cells. R means the ratio of green to red fluorescence intensity of each cell (*I*_*green*_/*I*_*red*_). (**A**) ROIs in normal HeLa cells; (**B**) ROIs in 60 μM chloroquine-stimulated HeLa cells; (**C**) ROIs in 5 μM dexamethasone-stimulated HeLa cells.

Finally, to establish a fully quantitative method for determining intercellular pH values, HeLa cells were treated with both **Rh-SA2** and the H^+^/K^+^ ionophore nigericin, which was applied to homogenize the endocellular pH with that of the surrounding medium. As exhibited in Fig. 6A, the green fluorescence emitted from the phenoxide unit in **Rh-SA2** enhances within the cells as the pH change from 5.0 to 7.5. This increase in green fluorescence intensity is paired with a corresponding decrease of fluorescence in the red channel from the proton-sensing rhodamine group. Gratifyingly, the ratio of green to red fluorescence intensity (R) displayed a specific pH-dependence with a good linearity over the pH range from 5.0 to 7.5 (Fig. 6B). This linear correlation allows for the relatively precise quantitative determination of pH values in various unique endocellular microenvironments. Toward this end, the endocellular pH changes in HeLa cells caused by stimulation with either chloroquine or dexamethasone were quantitatively measured (Fig. 6C). Taking into consideration that pH values vary significantly within living cells, we choose several ROIs in normal cells, chloroquine-stimulated cells, and dexamethasone-stimulated cells (which are all alive), respectively, and calculated the corresponding pH values from the ratio of fluorescence intensity in the green to red channels. The calculated results are displayed in Table S2. A detailed analysis of fluorescent intensity ratios is shown in Fig. 7. The red spots in the normal cells (ROI 1, 2, & 5 in a_2_ of Fig. 6C), have high acidic pH values (Table S2a, pH ~ 5.15). As demonstrated above, these regions represent the acidic vesicular lysosome organelles. The pH values of some orange spots (ROI 3 and 4 in a_2_ of Fig. 6C) are a litter higher but still weakly acidic (Table S2a, pH ~ 5.53). Upon stimulating with chloroquine, there are hardly any red spots visible in the image; the pH values of all ROIs rise to above 7.30. This outcome provided by **Rh-SA2** gives concrete evidence that chloroquine affects an increase in lysosomal pH. Meanwhile, a change in lysosomal topology was observed when the HeLa cells were exposed to dexamethasone: the red spots were more accumulated compared with normal cells, and their pH values were all less than 5.1. However there are some orange spots just like ROI 3 in c_2_ of Fig. 4C, in which the pH value was calculated as 5.61. These results verify that dexamethasone can lead to the acidification of living cells by gathering the lysosomes, which can further induce apoptosis. Combined, this quantitative data and corresponding results demonstrate that **Rh-SA2** not only has the ability to measure the pH values of normal cells and abnormal ones (alive, not fixed), but also has the potential for researching the effects of relevant drugs on endocellular pH throughout the cell.

### Optical stability and tissue imaging of Rh-SA2

In addition, the optical stability of **Rh-SA2** was also examined. The changes in the fluorescent images over a 300 s time period with an interval of 60 s was monitored at pH 4.0, 6.0, and 8.0, respectively (Fig. S8). The fluorescence intensity of both channels of **Rh-SA2** is still relatively stable over this 300 s at each pH value, indicating that **Rh-SA2** has good optical stability. This is particular helpful for real-time imagine of living cells.

**Figure 8:**
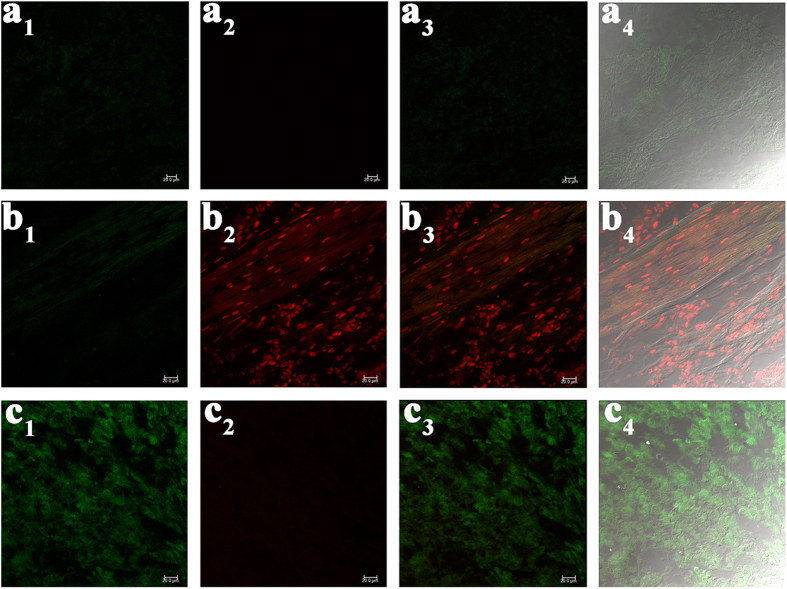
Confocal microscopy imaging of a frozen rat tumor slice. The first row: the tumor tissue stained with 10 μM **Rh-SA2**; the second row: followed by treatment with pH 5.0 PBS (10 mM); the third row: followed by treatment with pH 7.0 PBS (10 mM). The images of green (a_1_, b_1_, and c_1_) and red channels (a_2_, b_2_, and c_2_) were collected at 420–530 nm (λ_ex_ 405 nm) and 565–650 nm (λ_ex_ 550 nm), respectively. The whole tumor tissue was cultured with 10 μM **Rh-SA2** for 2 h, and then it was cut from one side.

Eventually, to further enlarge the application of **Rh-SA2**, a tissue imaging experiment was conducted (Fig. 8). The slice of mouse tumor stained with **Rh-SA2** only has weak fluorescence in the green channel (a_1_–a_4_) and essentially no fluorescence in the red channel. Following treatment with a pH 5.0 PBS buffer solution, however, the fluorescence of the red channel became much stronger (b_1_–b_4_). Alternatively, the green channel exhibited much stronger fluorescence following treatment with a pH 7.0 PBS buffer solution (c_1_–c_4_). These data suggest that **Rh-SA2** is capable to penetrate into and stain tissue, in addition to being a two-color (green and red) ratiometric pH-based imaging agent.

## Conclusion

In conclusion, we have developed an optimal pH probe **Rh-SA2** which possesses dual sensitive units for both acidic and basic environments. *In vitro*, **Rh-SA2** exhibits great sensitivity and selectivity toward various pH values within the scope endocellular pH. *In vivo*, **Rh-SA2** can not only offer a veracious and comprehensive pH map for both normal and abnormal cells, but it also can provide a quantitative measurement of cellular pH changes under drug-associated stimuli. This may be useful in investigating endocellular pH regulation mechanisms of drug action. Additionally, the successful application toward frozen tumor slices suggests that **Rh-SA2** has the potential for further studies in living tissues. More importantly, we have demonstrated a simple but effective pathway to construct a highly sensitive pH probe for the accurate mapping and quantification of endocellular pH values. The “double insurance” provided by our dual-channel probe **Rh-SA2** provides a unique, sensitive, accurate, and precise molecular tool to probe pH values in the complicated endocellular milieu.

## Method

### Reagents and chemicals

Unless otherwise noted, materials were obtained from commercial suppliers and were used without further purification. All solvents were dried according to standard methods prior to use. All of the solvents were either HPLC or spectroscopic grade in the optical spectroscopic studies. LysoTracker green DND-26 and nigericin were purchased from Invitrogen.

Birtton-Robison (B-R) buffer solutions consisting of 40 mM boric acid, 40 mM phosphoric acid, 40 mM acetic acid, and 20 mM sodium hydroxide were used for tuning pH values. All samples for fluorescence experiments were performed in different pH B-R buffer solution for 30 min before measurement. High K^+^ buffer solutions contain 30 mM NaCl, 120 mM KCl, 1 mM CaCl_2_, 0.5 mM MgSO_4_, 1 mM NaH_2_PO_4_, 5 mM glucose, 20 mM HEPES, 20 mM NaOAc, and 10.0 μM nigericin were used for changing endocellular values.

All experiments were performed in compliance with the relevant laws and institutional guidelines. All animal procedures were performed following the protocol approved by the Institutional Animal Care and Treatment Committee of Sichuan University (Chengdu, P.R. China). All of the mice were treated humanely throughout the experimental period.

### The pKa values of Rh-SA2

The individual pKa values for the rhodamine and phenolic units in each probe were estimated from the changes in fluorescence intensity at various pH values by using the relationship, log[(*I*_*max*_ − *I*)/(*I* − *I*_*min*_)] = pH − pKa, where *I*_*max*_, *I*_*min*_, and *I* are the maximum, minimum, and observed fluorescence intensity at a given pH, respectively^32^.

### Cell culture and imaging

HeLa cells were cultured in Dulbecco’s modified Eagle medium (DMEM) containing 10% fetal bovine serum and 1% antibiotic–antimycotic at 37 °C in a 5% CO_2_/95% air incubator. Fluorescence imaging, cells (4 × 10^3^ per well) were passed on confocal dishes and incubated for 24 h. Immediately before the staining experiments, the cells were washed twice with PBS (10 mM).

### Colocalization experiments in HeLa and HepG2 cells

HeLa and HepG2 cells were incubated with 1 μM LysoTracker Green and 5 μM **Rh-SA2** for 30 min at 37 °C, respectively. Then each dish was washed with PBS (10 mM) 3 times, and the resulting cells were analyzed with a confocal fluorescence microscope (Leica SP8). LysoTracker Green (the green emission) in 510–540 nm was collected using an excitation wavelength of 488 nm, **Rh-SA2** (the red emission) in 565–650 nm was collected using an excitation wavelength of 552 nm.

### Intracellular pH calibration

All the HeLa cells were incubated with 5 μM **Rh-SA2** for 30 min at 37 °C, respectively. Then each dish was washed with PBS (10 mM) 3 times, and the resulting cells were incubated with high K^+^ buffer at various pH values (pH 5.0, 5.5, 6.0, 6.5, 7.0, and 7.5) in the presence of 10.0 μM of nigericin. The fluorescence images were measured with a confocal microscope.

### Drug (chloroquine and dexamethasone) stimulus

All the HeLa cells were incubated with 5 μM **Rh-SA2** for 30 min at 37 °C, respectively. Then each dish was washed with PBS (10 mM) 3 times, and the resulting cells were incubated with various concentrations of chloroquine (30, 60, 120 μM) and dexamethasone (2.5, 5, 10 μM). Then, the fluorescence images were measured with a confocal microscope.

### Cytotoxicity

Toxicity toward HeLa cells and HepG2 cells was determined by Cell Counting Kit-8 (CCK-8; Dojindo Laboratories, Kumamoto, Japan) following literature procedures^38^. Briefly, about 9000 cells per well were seeded in 96-well plates and cultured for 24 h. After removing the old medium, HeLa cells and HepG2 cells were incubated with 2.5 μM and 5 μM Rh-SA2 for another 24 h. The medium was replaced by 100 mL fresh medium containing 10 mL CCK-8 and the plates were incubated at 37 °C for 2 h. Then, the absorbance at a wavelength of 450 nm of each sample was measured using an ELISA plate reader (BioRad, imark). Cellular viability (%) was obtained according to the manufacturer’s instruction.

### Preparation and staining of tumor tissue slices

The tumor was stripped from a nude mouse and, after washing with PBS (10 mM, pH 7.4) twice, it was cultured in 10 μM Rh-SA2 (PBS solution) at 37 °C for 2 h in a 5% CO_2_/95% air incubator. The resulting tumor was then washed with PBS (10 mM, pH 7.4) once, and a side of the tumor tissue was cut flat using a vibrating-blade microtome. The slices were fixed and divided into three groups, one as the control group for confocal microscopy imaging immediately, another group was followed by treatment with pH 5.0 PBS (10 mM), and the final group was followed by treatment with pH 7.0 PBS (10 mM).

## Additional Information

**How to cite this article**: Yu, K.-K. *et al.* A single design strategy for dual sensitive pH probe with a suitable range to map pH in living cells. *Sci. Rep.* **5**, 15540; doi: 10.1038/srep15540 (2015).

## Supplementary Material

Supplementary Information
