# Supplementary material for: A single design strategy for dual sensitive pH probe with a suitable range to map pH in living cells
Source: Sci Rep. 2015 Oct 21;5:15540. doi: 10.1038/srep15540 (PMC4614390; doi:10.1038/srep15540)
Supplement: Supplementary Information [file srep15540-s1.pdf]

# *Supporting Information*

## A single design strategy for dual sensitive pH probe with a suitable range to map pH in living cells

Kang-Kang Yu, Ji-Ting Hou, Kun Li,* Qian Yao, Jin Yang, Ming-Yu Wu, Yong-Mei Xie and Xiao-Qi Yu*

**Contents**

1. Apparatus & Synthesis of various compounds
2. Fluorescence and UV-Vis Spectroscopy
   Figure S1
   Figure S2
   Figure S3
3. p$K_a$ values of **Rh-SA2**
   Figure S4
4. Interferences of **Rh-SA2**
   Figure S5
5. Cytotoxicity and Cells Imaging
   Figure S6
   Figure S7
   Figure S8
   Table S1
   Table S2
6. Product Analysis
   $^{1}$H NMR spectra of **Rh-SA1/2/3** in $CDCl_3$
   $^{13}$C NMR spectra of **Rh-SA1/2/3** in $CDCl_3$
   ESI-MS of **Rh-SA1/2/3**
7. Reference

**Apparatus.** $^{1}$H NMR, $^{13}$C NMR spectra were measured on a Brüker AM400 NMR spectrometer. Proton chemical shifts (δ) of NMR spectra were given in ppm relative to internals reference TMS (1H, 0.00 ppm). ESI-MS and HRMS spectral data were recorded on a Finnigan LCQ$^{DECA}$ and a Brüker Daltonics Bio TOF mass spectrometer, respectively. All pH measurements were performed with a pH-3c digital pH-meter (Chengdu Fang Zhou Device Works, Chengdu, China) with a combined glass-calomel electrode. Fluorescence emission spectra were obtained using FluoroMax-4 Spectrofluorophotometer (HORIBA Jobin Yvon) at 298 K.

**Synthesis of various compounds.** **Rh-EDA** was synthesized according to our previous work.[1]

**Rh-SA1**: To a solution of **Rh-EDA** (242 mg, 0.5 mmol) in absolute methanol (20 ml) was added salicylaldehyde (1. 4 ml, 1 mmol) in one portion and the resulting mixture was stirred at room temperature for 7 hours. The solvent then was distilled *in vacuo*, and the resulting precipitate was purified by column chromatography to give **Rh-SA1** (216 mg, 73.4%). $^{1}$H NMR (400 MHz, $CDCl_3$) δ 8.09 (s, 1H), 7.97 (m, 1H), 7.46 (d, *J* = 5.6, 3.1 Hz, 2 H), 7.17 (m, 2H), 6.91 (d, *J* = 8.2 Hz, 1H), 6.86(m, 1H), 6.44 (d, *J* = 12.5, 5.7 Hz, 4H), 6.28 (d, *J* = 2.6 Hz, 2H), 3.51 (m, 12H), 1.28 (t, *J* = 7.1 Hz, 3H), 1.19 (t, *J* = 7.0 Hz, 12H). $^{13}$C NMR (100 MHz, $CDCl_3$) δ 168.3, 166.0, 161.1, 153.6, 153.4, 148.9, 132.5, 132.0, 131.1, 128.8, 128.0, 123.8, 122.8, 118.8, 118.3, 117.0, 108.1, 105.5, 97.8, 65.0, 57.1, 44.4, 40.9, 12.6. **Rh-SA1** *m/z* $[M+H]^+$ calcd 589.3173, found 589.3184; *m/z* $[M+Na]^+$ calcd 611.2993, found 611.2930.

**Rh-SA2**: To a solution of **Rh-EDA** (242 mg, 0.5 mmol) in absolute ethanol (20 ml) was added 4-hydroxyisophthalaldehyde (150 mg, 1 mmol), and the resulting mixture was stirred at room temperature for 7 hours. The solvent then was distilled *in vacuo*, and the resulting precipitate was purified by column chromatography to give **Rh-SA2** (150 mg, 48.7%). $^{1}$H NMR (400 MHz, $CDCl_3$) δ 14.22 (s, 1H), 9.80 (s, 1H), 8.03 (s, 1H), 7.92 (d, *J* = 5.8, 2.7 Hz, 1H), 7.79 (d, *J* = 8.7, 1.9 Hz, 1H), 7.66 (d, *J* = 1.8 Hz, 1H), 7.45 (d, *J* = 5.4, 3.3 Hz, 2H), 7.14 (m, 1H), 6.96 (d, *J* = 8.7 Hz, 1H), 6.40 (d, *J* = 7.6, 5.7 Hz, 4H), 6.23 (d, *J* = 8.9, 2.5 Hz, 2H), 3.49 (t, *J* = 6.5 Hz, 2H), 3.45(m, 2H), 3.33 (q, *J* = 7.0 Hz, 8H), 1.17 (t, *J* = 7.0 Hz, 12H). $^{13}$C NMR (100 MHz, $CDCl_3$) δ 189.9, 169.2, 168.4, 165.4, 153.4, 148.9, 134.9, 133.7, 132.6, 130.9, 128.8, 128.2, 127.2, 123.9, 122.9, 119.1, 117.9, 108.2, 105.4, 97.8, 65.0, 55.7, 44.4, 40.6, 22.6, 14.1, 12.6. **Rh-SA2** *m/z* $[M+H]^+$ calcd 617.3122, found 617.3136; *m/z* $[M+Na]^+$ calcd 639.2942, found 639.2903.

**Rh-SA3**: To a solution of **Rh-EDA** (242 mg, 0.5 mmol) in absolute ethanol (20 ml) was added 4-(diethylamino)-2-hydroxybenzaldehyde (150 mg, 1 mmol), and the resulting mixture was stirred at room temperature for 7 hours. The solvent then was distilled in vacuo, and the resulting precipitate was dissolved in small amounts of ethyl acetate. Controlled addition of petroleum ether to this solution resulted in the precipitation of a needle-like solid that was then recrystallized from diethyl ether to afford **Rh-SA3** (220 mg, 66.8%). $^{1}$H NMR (400 MHz, $CDCl_3$) δ 7.92 (q, *J* = 4.0 Hz, 1H), 7.73 (s, 1H), 7.43 (m, 2H), 7.08 (q, *J* = 4.0 Hz, 1H), 6.88 (d, *J* = 8.0 Hz, 1H), 6.42 (m, 4H), 6.25 (dd, *J* = 2.4, 8.8 Hz, 2H), 6.08 (m, 2H), 3.35 (m, 14H), 3.23 (t, *J* = 8.0 Hz, 2H), 1.17 (dd, *J* = 6.8, 12.4 Hz, 18H). $^{13}$C NMR (100 MHz, $CDCl_3$) δ 168.1, 166.7, 163.7, 153.61, 153.3, 151.6, 148.8, 132.8, 132.4, 131.1, 128.8, 127.9, 123.8, 122.8, 108.5, 108.1, 105.5, 102.9, 98.4, 97.8, 64.9, 54.5, 44.4, 41.2, 12.7. **Rh-SA3** calcd. $[M+H]^+$ *m/z* 660.3908, found 660.3897.

## Fluorescence and UV-Vis Spectroscopy

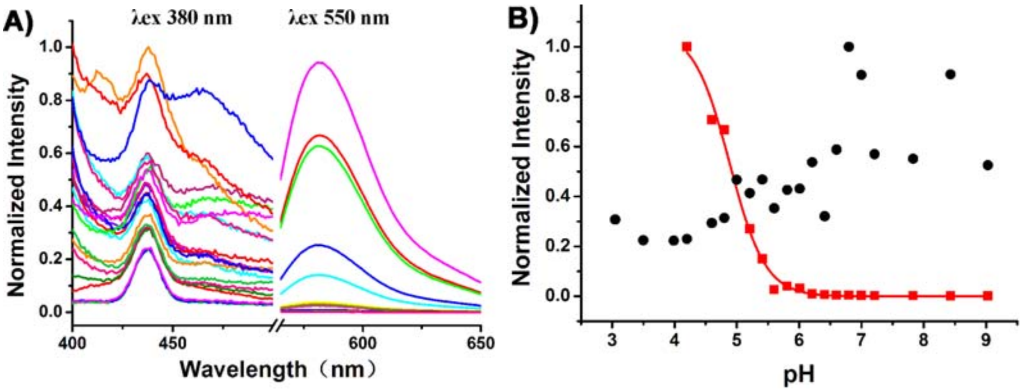


**Figure S1** A) Fluorescence emission spectral changes of **Rh-SA1** (5μM) in B–R buffer solution at different pH values, and the maximum emission intensity was measured at 440 nm ($\lambda_{ex}$ 380 nm) and 580nm ($\lambda_{ex}$ 550 nm). B) Plot of normalized fluorescence intensity at 440 nm (black) and 580nm (red) as a function of pH for **Rh-SA1**. pH 3.05, 3.50, 3.99, 4.20, 4.60, 4.80, 5.00, 5.21, 5.41, 5.60, 5.81, 6.01, 6.21, 6.41, 6.60, 6.81, 7.00, 7.21, 7.83, 8.42, and 9.08.

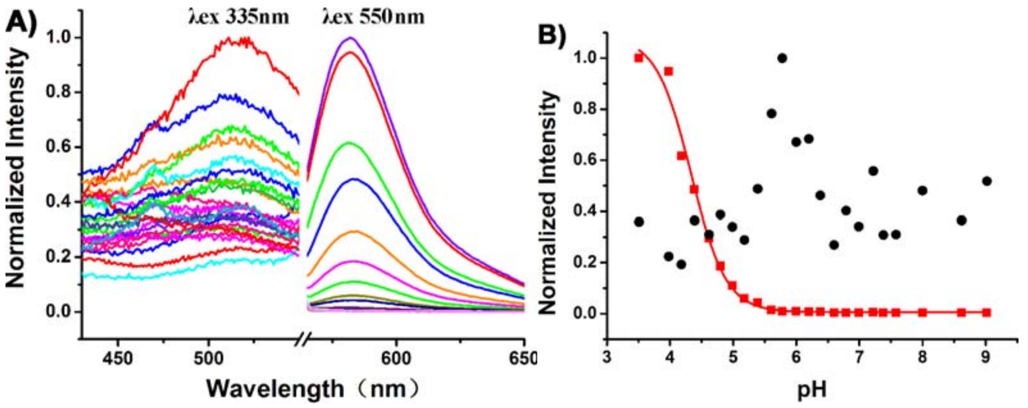


**Figure S2** A) Fluorescence emission spectral changes of **Rh-SA3** (5μM) in B–R buffer solution at different pH values, and the maximum emission intensity was measured at 510 nm ($\lambda_{ex}$ 335 nm) and 580nm ($\lambda_{ex}$ 550 nm). B) Plot of normalized fluorescence intensity at 510 nm (black) and 580nm (red) as a function of pH for **Rh-SA3**. pH 3.51, 3.98, 4.18, 4.39, 4.62, 4.80, 4.99, 5.18, 5.39, 5.61, 5.78, 6.01, 6.20, 6.38, 6.60, 6.79, 6.99, 7.22, 7.38, 7.58, 8.00, 8.62 and 9.02.

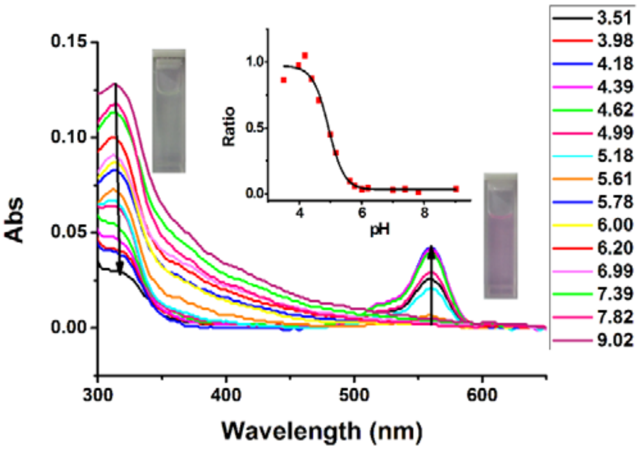


**Figure S3** UV-Vis absorption of **Rh-SA2** (5 μM) in B-R buffer at different pH values

## $pK_a$ values of Rh-SA2

Henderson-Hasselbach-type mass action equation: $pK_a = pH - \log [(I_{max}-I)/(I-I_{min})]$

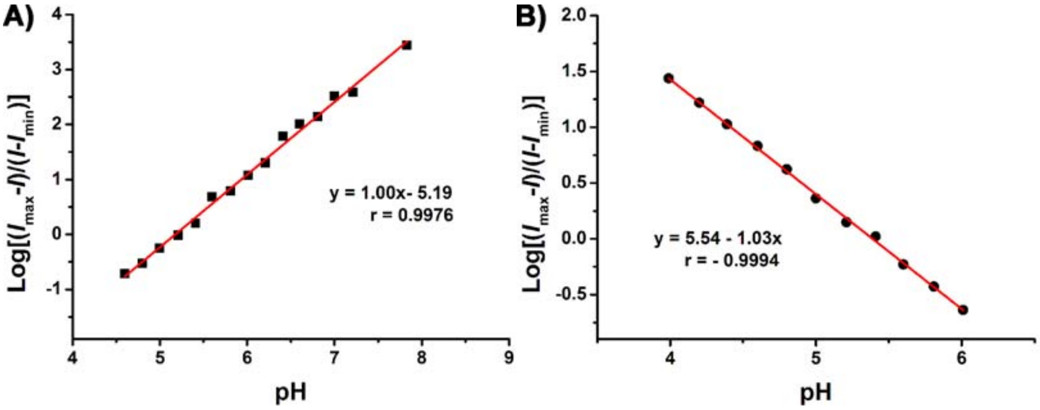


**Figure S4** A)The calculation for the $pK_a$ value of rhodamine unit of **Rh-SA2**, B) The calculation for the $pK_a$ value of phenol unit of **Rh-SA2**.

## Interferences of Rh-SA2

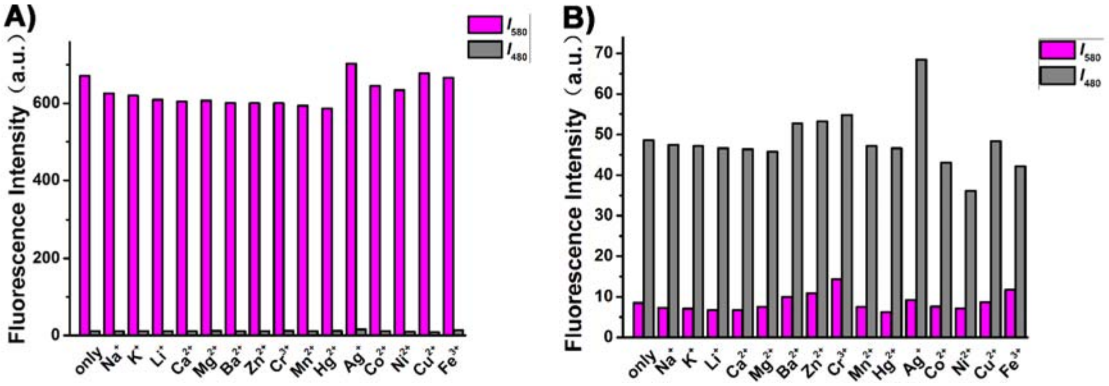

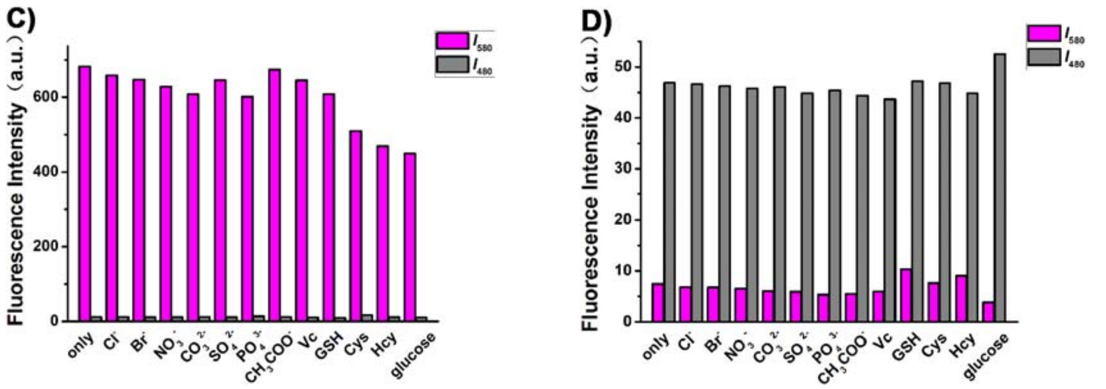


**Figure S5** Pink bars: Fluorescence response of **Rh-SA2** (5 μM) at 580 nm toward other competitive compounds in B-R buffer solution. Grey bars: selectivity of **Rh-SA2** (5 μM) for pH at 480 nm toward other selected interferences in B-R buffer solution. A) and C) pH 5.0 B-R solution, C) and D) pH 7.0 B-R solution. The concentration of interferences: anions and cations 500μM; GSH, Cys, and Hcy 3 mM, glucose 5mM.

## Cytotoxicity and Cells Imaging

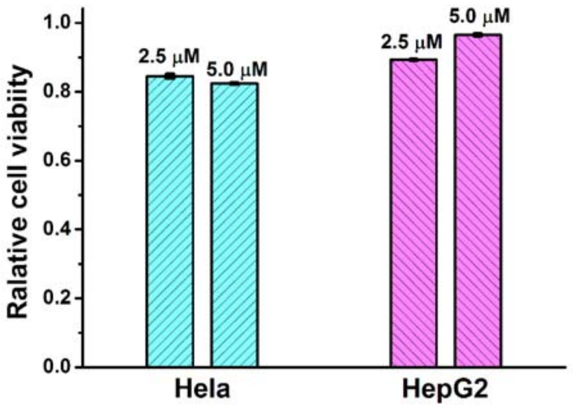


**Figure S6** Cytotoxicity of **Rh-SA2** on Hela and HepG2 cells.

## Colocalization experiments

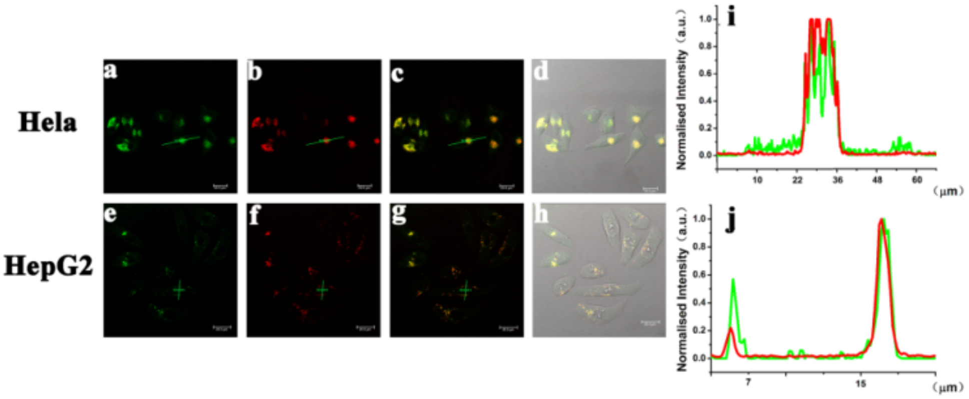


**Figure S7.** Colocalization experiments in HeLa and HepG2 cells. The cells were incubated with 5μM **Rh-SA2** and 1μM LysoTracker Green (LTG) for 30 min at 37℃. Cell images were then collected at 510–540 nm for the green channel a and e of LTG ($\lambda_{ex}$ 488 nm) and 565–650 nm for the red channel b and f of **Rh-SA2** ($\lambda_{ex}$ 550 nm). i)

Intensity profile of ROI across Hela cells; j) Intensity profile of ROI across HepG2 cells. (green line – green channel, red line – red channel).

Pearson's correlation was calculated by the Image-Pro Plus software.

## Time course confocal microscopy images of HeLa cells

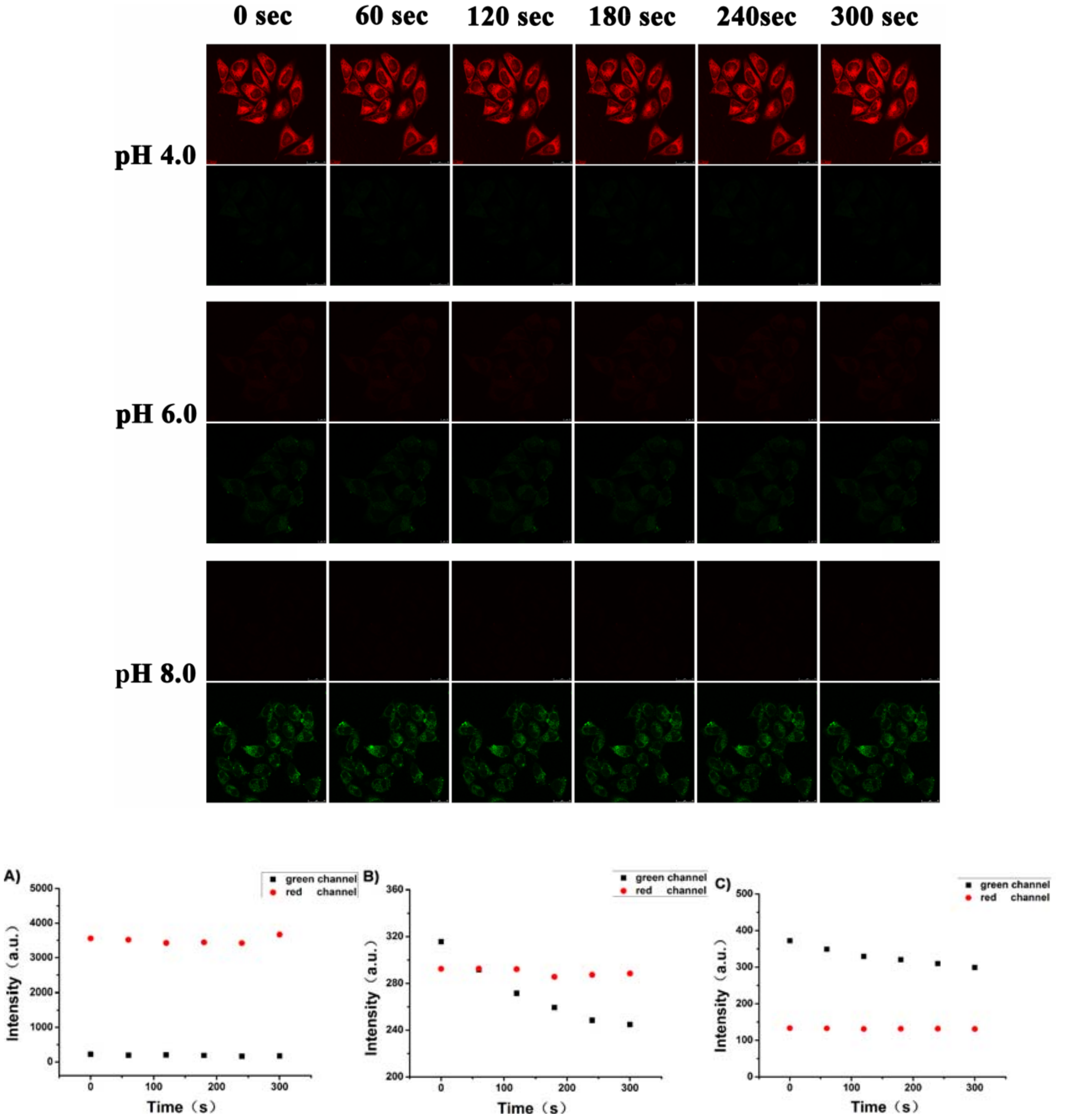


**Figure S8** Time course confocal microscopy images of HeLa cells clamped at pH 4.0, 6.0, and 8.0. **Rh-SA2** 5 μM, and all the cell images were collected at 420–530 nm for the green channel (bottom, $\lambda_{ex}$ 405 nm) and 565–650 nm for the red channel (top, $\lambda_{ex}$ 550 nm). Fluorescence spectra analysis of the HeLa cells at A) pH 4.0, B) pH 6.0, and C) pH 8.0. (black spots: green channel; red spots: red channel ).

| compound | | φ(rhodamine) | φ(salicylaldehyde) |
|---|---|---|---|
| Rh-SA1 | pH=3.98 | 0.379 | 0.005 |
| | pH=6.60 | 0.005 | 0.005 |
| Rh-SA2 | pH=3.98 | 0.343 | 0.082 |

| | | | |
|---|---|---|---|
| | pH=6.99 | 0.015 | 0.023 |
| Rh-SA3 | pH=3.98 | 0.013 | 0.006 |
| | pH=6.00 | 0.015 | 0.005 |

**Table S1.** The quantum yields of **Rh-SA2** at pH 4.0 and 7.0

**a**

| Hela Cell | ROI 1 | ROI 2 | ROI 3 | ROI 4 | ROI 5 |
|---|---|---|---|---|---|
| R | 0.24827 | 0.23674 | 0.64375 | 0.50628 | 0.26801 |
| pH | 5.15 | 5.14 | 5.62 | 5.45 | 5.17 |

**b**

| Hela Cell | ROI 1 | ROI 2 | ROI 3 | ROI 4 | ROI 5 |
|---|---|---|---|---|---|
| R | 2.30298 | 2.09294 | 2.37782 | 2.07013 | 2.13223 |
| pH | > 7.50 | 7.32 | > 7.50 | 7.30 | 7.37 |

**c**

| Hela Cell | ROI 1 | ROI 2 | ROI 3 |
|---|---|---|---|
| R | 0.19343 | 0.20180 | 0.64335 |
| pH | 5.08 | 5.09 | 5.61 |

**Table S2.** The calculated pH values of ROI from Figure 3C

## 8. Product Analysis

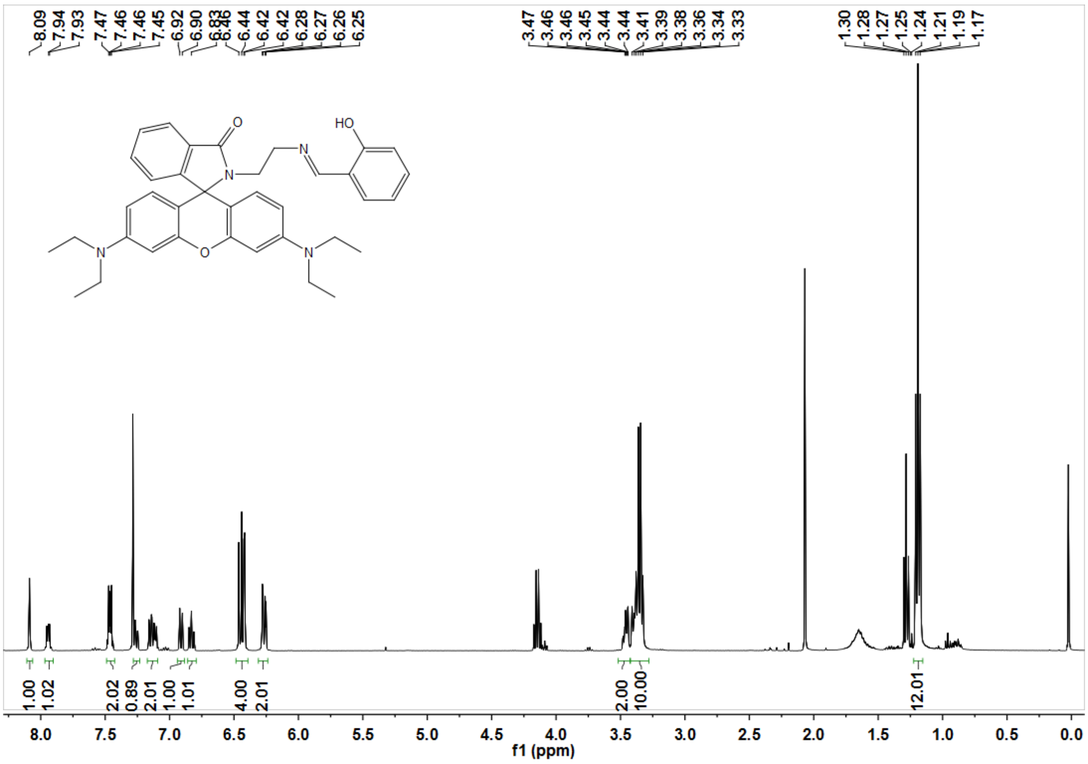


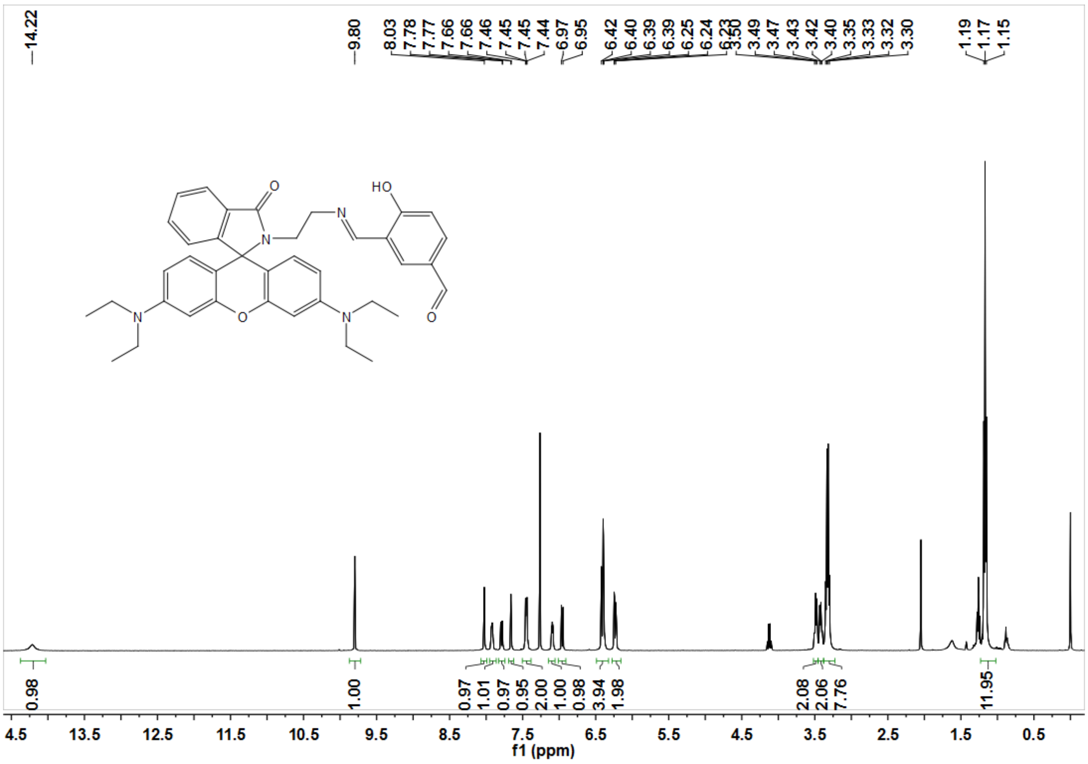

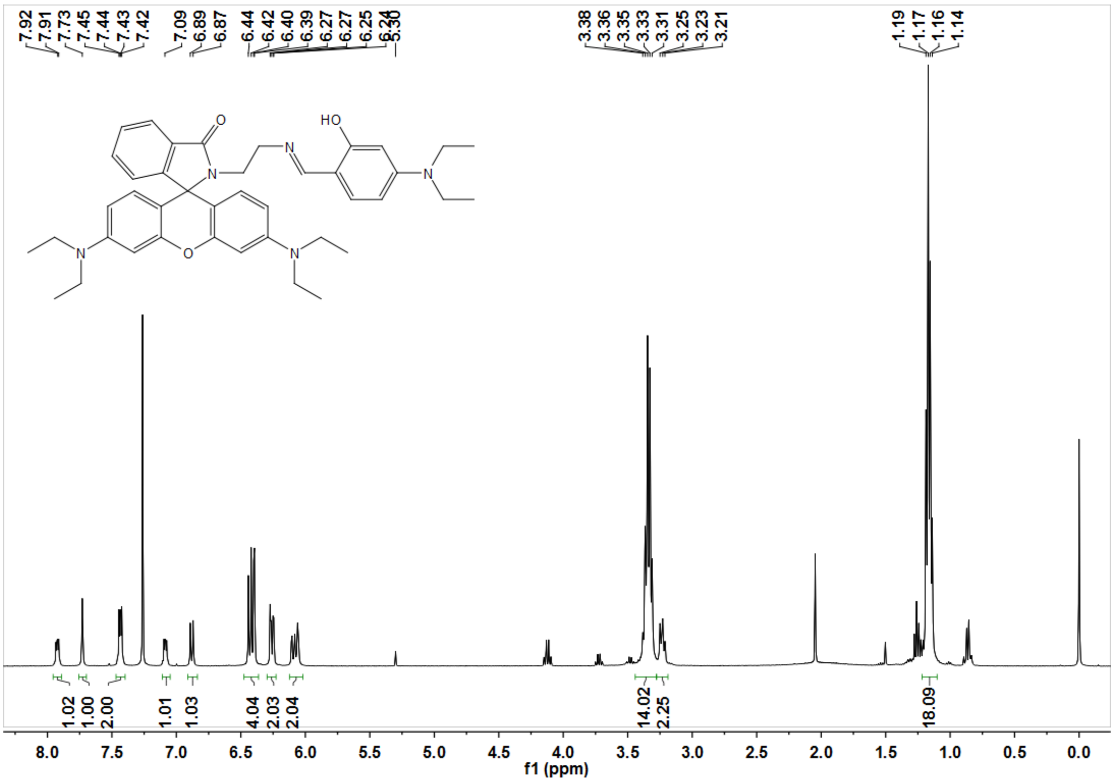
7.92 7.91 7.73 7.45 7.44 7.43 7.42 7.09 6.89 6.87 6.44 6.42 6.40 6.39 6.27 6.27 6.25
3.38 3.36 3.35 3.33 3.31 3.25 3.23 3.21
1.19 1.17 1.16 1.14
HO
O
N
N
O
1.02 1.00 2.00 1.01 1.03 4.04 2.03 2.04 14.02 2.25 18.09
8.0 7.5 7.0 6.5 6.0 5.5 5.0 4.5 4.0 3.5 3.0 2.5 2.0 1.5 1.0 0.5 0.0
f1 (ppm)

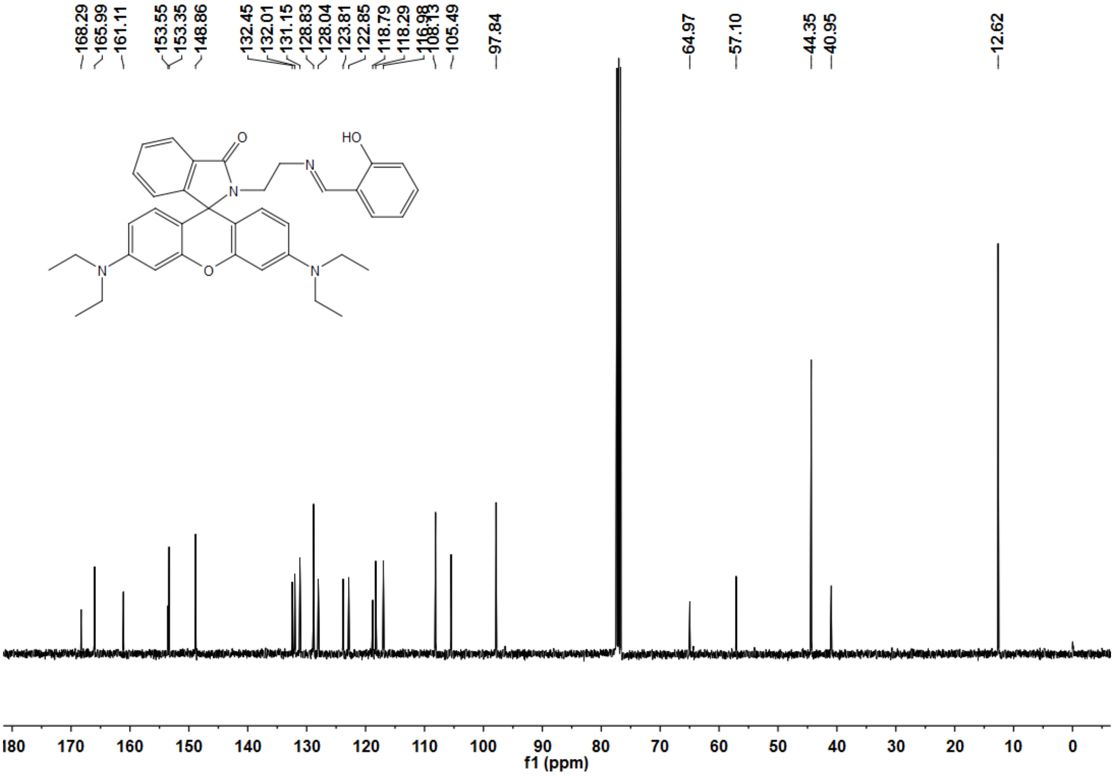
168.29 165.99 161.11 153.55 153.35 148.86 132.45 132.01 131.15 128.83 128.04 123.81 122.85 118.79 118.29 105.49 97.84
64.97 57.10 44.35 40.95 12.62
HO
O
N
N
O
180 170 160 150 140 130 120 110 100 90 80 70 60 50 40 30 20 10 0
f1 (ppm)

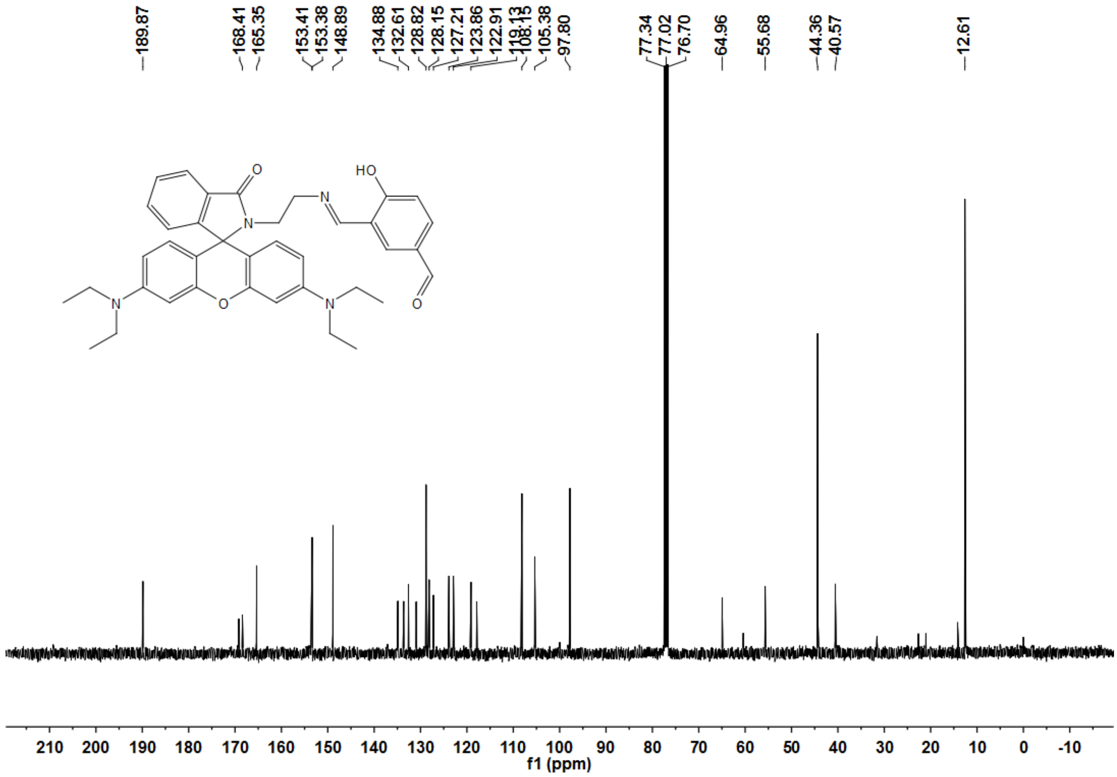
189.87
168.41
165.35
153.41
153.38
148.89
134.88
132.61
128.82
128.15
127.21
123.86
122.91
119.13
108.15
105.38
97.80
77.34
77.02
76.70
64.96
55.68
44.36
40.57
12.61
HO
O
N
O
N
N
O
N
210 200 190 180 170 160 150 140 130 120 110 100 90 80 70 60 50 40 30 20 10 0 -10
f1 (ppm)

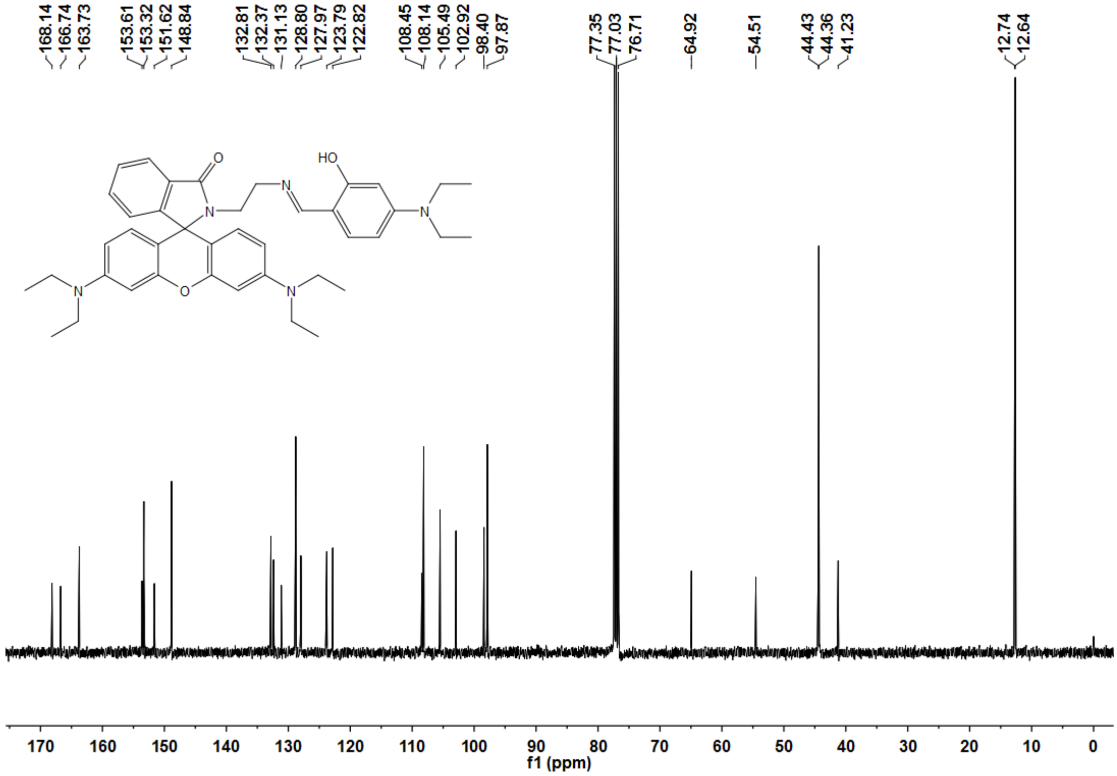
168.14
166.74
163.73
153.61
153.32
151.62
148.84
132.81
132.37
131.13
128.80
127.97
123.79
122.82
108.45
108.14
105.49
102.92
98.40
97.87
77.35
77.03
76.71
64.92
54.51
44.43
44.36
41.23
12.74
12.64
HO
O
N
N
N
N
O
N
170 160 150 140 130 120 110 100 90 80 70 60 50 40 30 20 10 0
f1 (ppm)

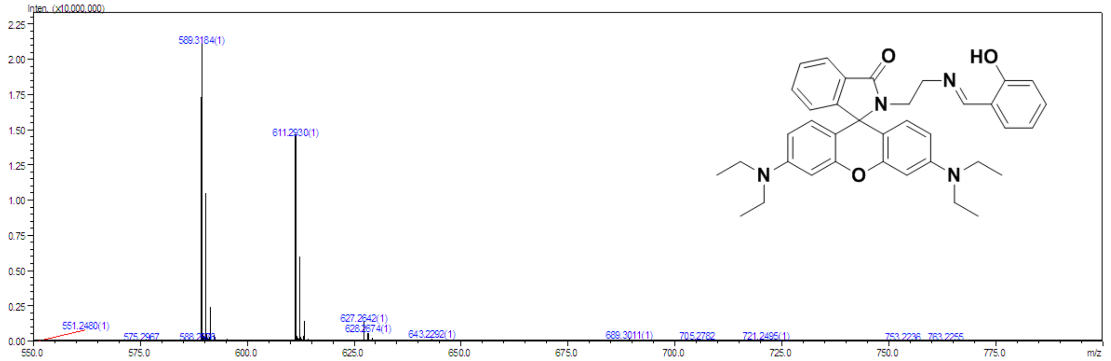


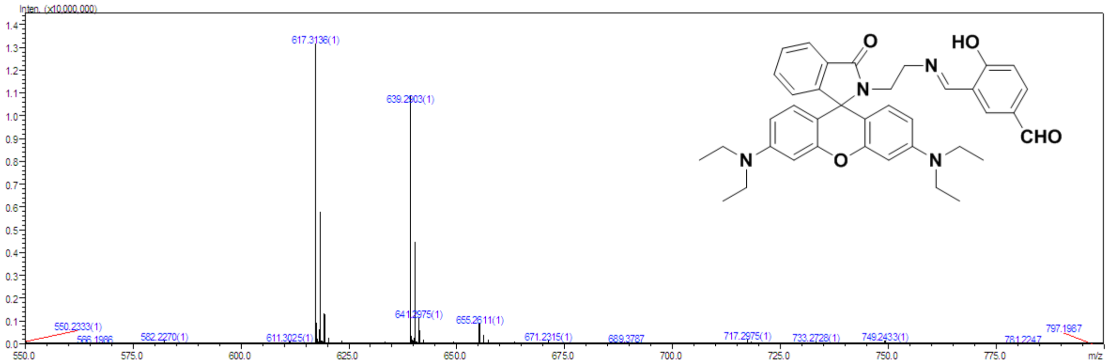


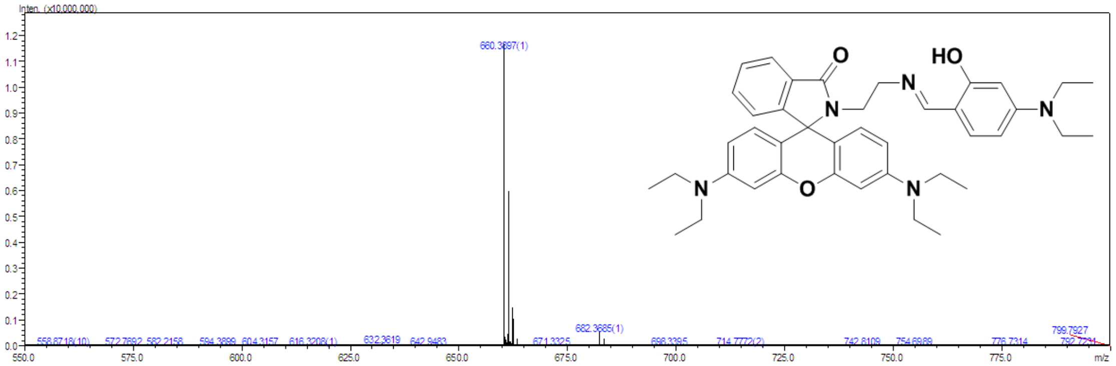


## Reference

1 K.-K. Yu, K. Li, J.-T. Hou, J. Yang, Y.-M. Xie and X.-Q. Yu, *Polym. Chem.*, 2014, **5**, 5804–5812; J.-T. Hou, M.-Y. Wu, K. Li, J. Yang, K.-K. Yu, Y.-M. Xie and X.-Q. Yu, *Chem. Commun.*, 2014, **50**, 8640-8643.
